# The association between mobility and asleep blood pressure alterations in frail and non-frail adults 80 years and older

**DOI:** 10.1038/s41371-026-01199-6

**Published:** 2026-08-19

**Authors:** Fernando G. Blauth, Fernando L. Scolari, Laís A. S. Vilar, Victor C. B. Pontes, Vanessa B. V. de Carvalho, Júlio C. Moriguti, Nereida K. Lima

**Affiliations:** 1 Division of Internal Medicine and Geriatrics, Department of Internal Medicine, Ribeirão Preto Medical School, University of São Paulo, Ribeirão Preto, Brazil; 2 Division of Cardiology, Hospital de Clínicas de Porto Alegre, Porto Alegre, Brazil

**Keywords:** Hypertension, Risk factors, Ageing

## Abstract

The influence of mobility on asleep blood pressure among adults aged 80 years and older, particularly considering frailty, remains poorly understood. This cross-sectional study aimed to examine the association between objectively measured mobility parameters and asleep blood pressure alterations in a frail and non-frail population aged 80 years and older. Seventy-one participants (mean age 85 ± 4 years; 33 non-frail and 38 frail) completed both ambulatory blood pressure monitoring (ABPM) and objective mobility assessment using actigraphy. Baseline characteristics were similar between groups, except for a higher prevalence of hypertension and greater comorbidity burden in frail individuals. Mobility measures reflected a more sedentary profile in the frail group. In the total population, sedentary time, walking time, steps/day, cadence, and energy expenditure (METs-h) showed significant linear associations with asleep SBP and/or SBP dipping. Higher sedentary time correlated with elevated asleep SBP and reduced SBP dipping, whereas greater walking time, steps/day, cadence, and METs-h were protective. Among frail individuals, cadence was the only parameter consistently associated with both asleep SBP and dipping. In multivariable logistic regression adjusted for age, hypertension, and frailty status, higher cadence remained independently protective against elevated asleep SBP (OR 0.56, 95% CI 0.39–0.80) and non-dipping pattern (OR 0.87, 95% CI 0.79–0.95). In conclusion, mobility parameters were closely associated with asleep blood pressure alterations, specifically elevated asleep SBP and changes in the dipping pattern, among frail and non-frail adults aged 80 years and older. Cadence emerged as the most robust and clinically applicable measure.

**Figure:**
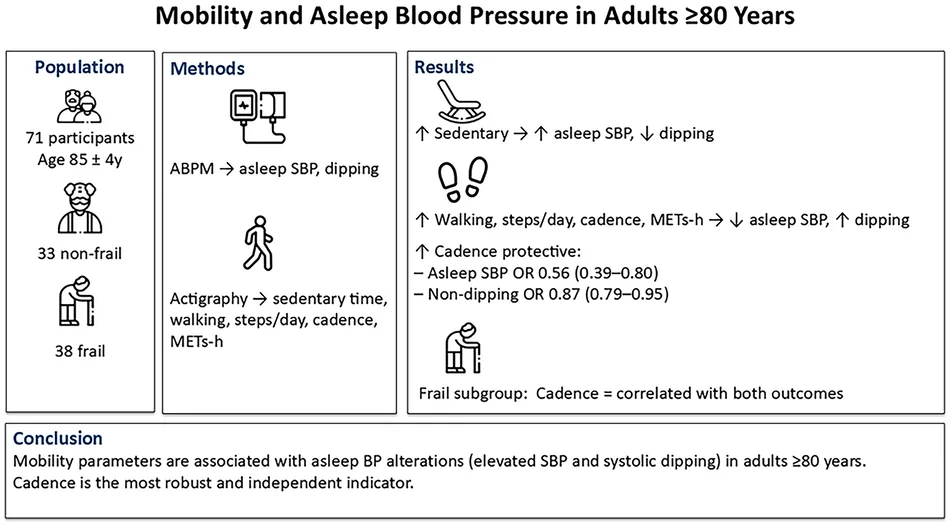

## Introduction

Despite strong evidence supporting antihypertensive treatment in adults aged 80 and above, cardiovascular risk assessment in this population remains challenging, given that intrinsic capacity independently influences outcomes beyond chronological age [1–5]. Elevated asleep systolic blood pressure (SBP) and the lack of a physiological nocturnal decline in blood pressure, referred to as a non-dipping pattern, have proven to be useful indicators of cardiovascular risk in older adults. They are associated with higher mortality and adverse clinical outcomes, including cognitive impairment and cardiovascular events, and provide prognostic information beyond conventional blood pressure measurements such as office, awake, and 24-h blood pressure [6–8].

Frailty plays a pivotal role in understanding this population, as it has been linked to a higher risk of non-dipping pattern, independent of 24-h blood pressure levels or the presence of hypertension [9]. However, frailty evaluation in medical gerontology is diverse [10]. There are many different scores, with distinct cutoff points and sometimes diverging definitions, hindering clinical applicability [11]. Therefore, more precise and objective parameters closely linked to frailty are needed to assess cardiovascular risk in adults aged 80 years and older.

Mobility parameters have been associated with variations in blood pressure pattern with greater influence on sleeping periods [12]. Previous studies have shown that more physically active individuals are more likely to exhibit a normal systolic dipping pattern during sleep [13]. Although most definitions of frailty include mobility-related parameters, no study to date has examined the influence of these parameters on asleep blood pressure patterns in frail populations.

The aim of this study is to investigate the association between mobility and asleep blood pressure alterations, specifically elevated asleep SBP and changes in SBP dipping, among frail and non-frail adults aged 80 years and older, using a cross-sectional design.

## Materials and methods

### Participants

This is a sub-analysis of a previously published cross-sectional study [9]. From March 2019 through April 2021, consecutive patients aged 80 years and older attending a geriatric outpatient clinic of the University of São Paulo, located in Ribeirão Preto, São Paulo, Brazil, were screened for eligibility. Frailty status was assessed during the initial screening, together with the other eligibility criteria. Individuals classified as robust or frail, who did not meet any exclusion criteria, were considered eligible and invited to participate. Pre-frail individuals were excluded a priori because pre-frailty represents an intermediate and more heterogeneous stage and, in this reduced sample, contrasting robust and frail individuals was considered a more appropriate strategy to maximize discrimination between clearly distinct frailty phenotypes. We excluded patients who were bedridden or unable to walk, who had any condition that could impair 24-h blood pressure measurements (arrhythmia or advanced cognitive disorders), or who were using continuous positive airway pressure (CPAP) devices during sleep.

A complete medical history, including comorbidities and medications in use, was collected in all individuals through direct interview and medical records review. Physical examination was performed to assess weight, height, and abdominal circumference. Previous hypertension was defined as any of the following: medical record confirmation, use of any anti-hypertensive medication, systolic blood pressure ≥140 mmHg or diastolic ≥90 mmHg on two different occasions [14]. Following an initial comprehensive evaluation, the individuals were submitted to 24-h ambulatory blood pressure monitoring (ABPM) and 7-day mobility measurement.

Due to the outbreak of the COVID-19 pandemic, data collection was suspended during local restrictions. Appropriate safety measures were taken when the study was restarted, and special care was taken to ensure that all enrolled subjects were asymptomatic at the time of analysis. This study was approved by an independent Ethics Committee (protocol no. 3.500.611) and written informed consent was obtained from all individuals or their legal surrogates.

### Frailty assessment

Frailty was analyzed according to the phenotype model proposed by Fried et al., where individuals are classified as robust, pre-frail, or frail if they fulfill none, one or two, and more than two of the five proposed criteria, respectively [15].

The criteria analyzed included slow gait, which was measured by walking speed over a distance of 4.6 m. Reduced hand grip strength was assessed using a dynamometer (Jamar Hydraulic Hand Dynamometer) with three consecutive readings in the dominant hand. Unintentional weight loss was defined as a loss of ≥4.5 kg or ≥5% of total body weight in the preceding year. Exhaustion or fatigue was evaluated through self-reported responses to questions from the Center for Epidemiological Studies Depression Scale. Low physical activity was defined using the International Physical Activity Questionnaire (IPAQ) as engaging in less than ten consecutive minutes of any activity during the last week [16].

The reference values for these criteria were adjusted for height, sex, and body mass index (BMI) based on data from a local population study [17].

### Ambulatory blood pressure monitoring

Ambulatory blood pressure monitoring was performed during a 24-h period by an automated device (Spacelabs Medical model 90207, Issaquah, WA, USA). A proper cuff was used according to non-dominant arm size and circumference. The device was programed to obtain measures every fifteen minutes from 6 AM to 10 PM and every thirty minutes from 10 PM to 6 AM. Individuals were oriented to inform sleeping and waking time in a diary and maintain normal routine during analysis.

The exam was considered adequate if at least 75% of the measurements were valid, with at least 16 valid readings during daytime and 8 during nighttime. The patient´s diary was used to define awake and sleeping periods. In case these data were missing, sleeping period was considered from 10 PM to 6 AM.

Asleep blood pressure dipping was calculated as the percentage decline in systolic blood pressure from awake to asleep periods using the formula [(awake SBP – asleep SBP)/awake SBP]. Systolic dipping patterns were classified as follows: reverse dipping, <0%; non-dipping, 0 to <10%; normal dipping, 10 to 20%; and extreme dipping, >20%. For the main analyses, the non-dipping pattern was considered present when the systolic BP decline was <10%. The mean of all valid readings was used for the analysis, and the entire process of device installation, measurement acquisition, and data interpretation was conducted in accordance with local guidelines [18].

### Mobility assessment

Spontaneous mobility was assessed over a seven-day period using a thigh-worn accelerometry-based actigraph (activPAL, PAL Technologies Ltd., Glasgow, UK). The device, which weighs 15 grams, was attached to the mid-lateral region of the right thigh with a transparent hypoallergenic adhesive and continuously recorded posture and movement patterns during usual daily activities. Based on thigh inclination and acceleration, the device performs four measurements per second and classifies time spent sitting/lying, standing, and walking, and provides step-based ambulatory metrics. All individuals were oriented to maintain normal routine during analysis. If evaluation was interrupted or not completed by any motive the data was excluded from mobility analysis.

The variables considered for analysis were time spent at rest, in orthostasis, and walking, number of transfers, number of steps, cadence, and total energy expenditure [metabolic equivalent of task (METs)]. Time variables are presented as min/day. Cadence was defined as the number of steps per minute during walking. Unlike gait speed, which reflects the distance covered over time during a standardized walking test, cadence reflects habitual walking rhythm during free-living ambulation. METs-h was estimated using a validated equation [19].

### Statistical analysis

#### Descriptive statistics and group comparisons

The sample size was estimated based on the minimum number of participants required to detect a clinically relevant difference in asleep systolic blood pressure between frail and non-frail individuals. Calculations assumed a two-sided α of 0.05 and 80% power. An expected between-group difference of 15 mmHg and a standard deviation of 22 mmHg were considered, based on previous studies with similar methodology.

Continuous variables are presented as mean ± standard deviation (SD) or median with interquartile range (IQR), according to data distribution. Normality was assessed using the Shapiro-Wilk test. Comparisons between frail and non-frail individuals were conducted using the Student’s t-test for normally distributed variables or the Mann-Whitney U test for non-normally distributed data. Categorical variables are expressed as absolute frequencies and percentages, and comparisons were performed using the Pearson chi-square test.

### Correlation analysis

To assess the association between mobility parameters and asleep blood pressure variables (asleep systolic blood pressure and systolic blood pressure dipping), Pearson’s correlation coefficient (r) was calculated for normally distributed variables, and Spearman’s rank correlation coefficient (ρ) was applied for variables with non-normal distribution. For each correlation, both the coefficient and the corresponding p-value were reported. Analyses were performed for the total sample, as well as separately for frail and non-frail subgroups.

### Trend analysis across tertiles

Participants were stratified into tertiles based on each mobility parameter. To test for ordered differences in asleep systolic blood pressure and systolic dipping across mobility tertiles, the Jonckheere-Terpstra test was applied as a non-parametric test for trend. The expected direction of the trend (increasing or decreasing) for each mobility variable was defined based on clinical assumptions.

### Logistic regression

Logistic regression analyses were performed to evaluate the association between mobility and two outcomes: asleep systolic blood pressure (continuous) and non-dipping pattern (binary). A multivariate model was constructed to adjust for potential confounders. The covariates included for adjustment were age, hypertension and frailty. Results are presented as odds ratios (ORs) with 95% confidence intervals (95% CI).

### Software and visualization

All statistical analyses were conducted using SAS version 9.4 (SAS Institute, Cary, NC, USA) and SPSS version 25 (IBM Corp., Armonk, NY, USA). Graphs and data visualizations, including correlation plots, boxplots, and trend analyses, were generated using Python in Google Colab. A two-tailed p-value < 0.05 was considered statistically significant.

## Results

**Fig. 1:**
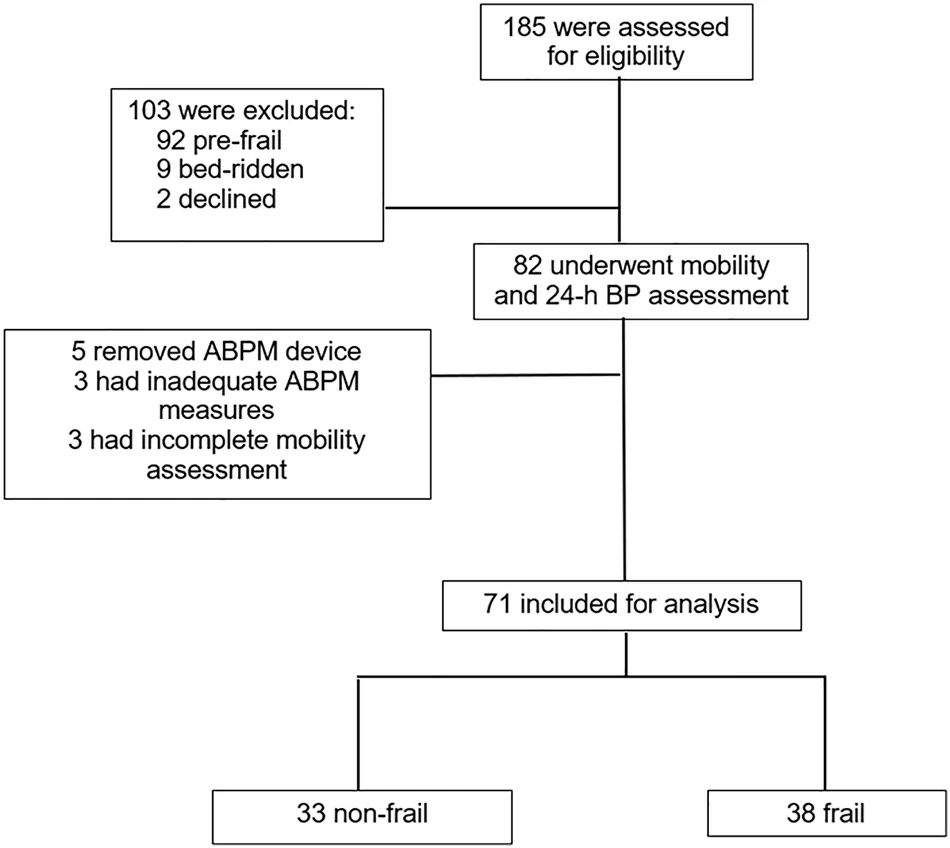
Study design. BP blood pressure, ABPM ambulatory blood pressure measurement.

A total of 185 individuals were assessed for frailty and 82 (44.3%) were eligible to participate. Five (6.1%) of the 82 individuals removed their ABPM device during analysis, three (3.6%) had inadequate measurements (one frail and two non-frail), and three had incomplete mobility assessment thus, a total of 71 individuals were included for analysis, 33 non-frail and 38 frail. The complete study design is shown in Fig. 1.

The mean age of the study population was 85 ± 4 years with a predominance of the female gender. Age, gender and other baseline characteristics were similar between groups [Table 1]. The number of comorbidities was higher in the frail group [4 (3–6) vs 7 (5–9); *p* < 0.01]. There was a higher prevalence of hypertension in the frail group [21 (63%) vs 34 (89%); *p* < 0.01], but the number of antihypertensive drugs did not differ [2 (0–2) vs. 1 (1–2); *p* = 0.21]. The prevalence of diabetes [7 (21%) vs. 13 (34%), *p* = 0.25] and dyslipidemia [18 (54%) vs. 17 (44%); *p* = 0.41] was similar between groups. According to ABPM, frail individuals exhibited a higher prevalence of the non-dipping pattern and a tendency toward elevated asleep SBP. Among frail participants, mobility measures reflected a more sedentary status, except for the number of transfers [Table 1]. Because this study is a sub-analysis of a previously published cross-sectional study, the detailed baseline 24-h, awake, and asleep ABPM values of frail and non-frail participants were not repeated here and have been reported elsewhere [9].

**Table 1:** Demographic characteristics of study participants with mobility measurements.

Variable	Total (*n* = 71)	Non-Frail (*n* = 33)	Frail (*n* = 38)	p-value
Age (years)	85 ± 4	85 ± 3	86 ± 4	0.30
*Gender*				
Female	49	20 (61%)	29 (76%)	0.15
*Ethnicity*				0.85
White	55	25 (76%)	27 (71%)	
Black	8	3 (9%)	5 (13%)	
BMI	27 ± 5	27 ± 4	27 ± 5	0.80
Numb. of Comorbidities	5 (4–7)	4 (3–6)	7 (5–9)	<0.01
Numb. of Antihypertensives	1 (1–2)	2 (0–2)	1 (1–2)	0.21
Hypertension	55	21 (63%)	34 (89%)	<0.01
Diabetes	20	7 (21%)	13 (34%)	0.22
Dyslipidemia	35	18 (54%)	17 (44%)	0.41
*Mobility*				
Sedentary time (min/day)	1110 ± 155	1024 ± 138	1184 ± 130	<0.01
Time in orthostasis (min/day)	324 ± 143	320 ± 105	223 ± 115	<0.01
Walking time (min/day)	56 ± 37	83 ± 30	33 ± 26	<0.01
Numb. Transfers	626 ± 194	661 ± 176	596 ± 206	0.15
Steps/day	3895(±2916)	6027 ± 2517	2043 ± 1740	<0.01
Cadence (steps/min)	64 ± 12	72 ± 8	57 ± 11	<0.01
METS-h	226 ± 9	232 ± 7	220 ± 6	<0.01

Data are mean ± SD, or median (P25-P75) for continous variables or n (%) for categorical variables.

*BMI* body mass index.

p value for the difference between frail and non-frail.

The sedentary time, time in orthostasis, walking time, number of steps per day, cadence and METS-h showed linear correlation with nocturnal blood pressure variables (asleep SBP and/or SBP dipping) in the total study population as shown in Table 2. Cadence showed the highest correlation with asleep SBP (r = −0.39, *p* < 0.01) and SBP dipping (r = 0.55, *p* < 0.01). In a subgroup restricted to frail individuals, cadence was the only parameter to show moderate correlation with both asleep SBP (r = −0.4, *p* = 0.01) and SBP dipping in the frail group (r = 0.34, *p* = 0.03) [Table 2].

**Table 2:** Linear correlation between mobility parameters and asleep blood pressure.

	Total population				Non-Frail				Frail			
	Asleep SBP		SBP Dipping		Asleep SBP		SBP Dipping		Asleep SBP		SBP Dipping	
Variable	r	p-value	r	p-value	r	p-value	r	p-value	r	p-value	r	p-value
Sedentary time (min/day)	0.11	0.33	−0.23	0.05	−0.11	0.51	−0.2	0.25	0.11	0.48	0.09	0.56
Time in orthostasis (min/day)	−0.1	0.4	0.15	0.22	0.09	0.6	0.16	0.35	−0.09	0.55	−0.14	0.39
Walking time (min/day)	−0.24	0.04	0.41	<0.01	−0.13	0.46	0.32	0.06	−0.15	0.36	0.13	0.4
Numb. Transfers	−0.18	0.11	0.17	0.15	−0.25	0.16	0.14	0.41	−0.09	0.56	0.09	0.56
Steps/day	−0.29	0.01	0.47	<0.01	−0.21	0.24	0.41	0.01	−0.24	0.14	0.19	0.23
Cadence (steps/min)	−0.39	<0.01	0.55	<0.01	−0.25	0.16	0.49	<0.01	−0.4	0.01	0.34	0.01
METS-h	−0.28	0.02	0.44	<0.01	−0.16	0.35	0.41	0.01	−0.22	0.17	0.11	0.5

*SBP* systolic blood pressure, *r* correlation coefficient.

**Fig. 2:**
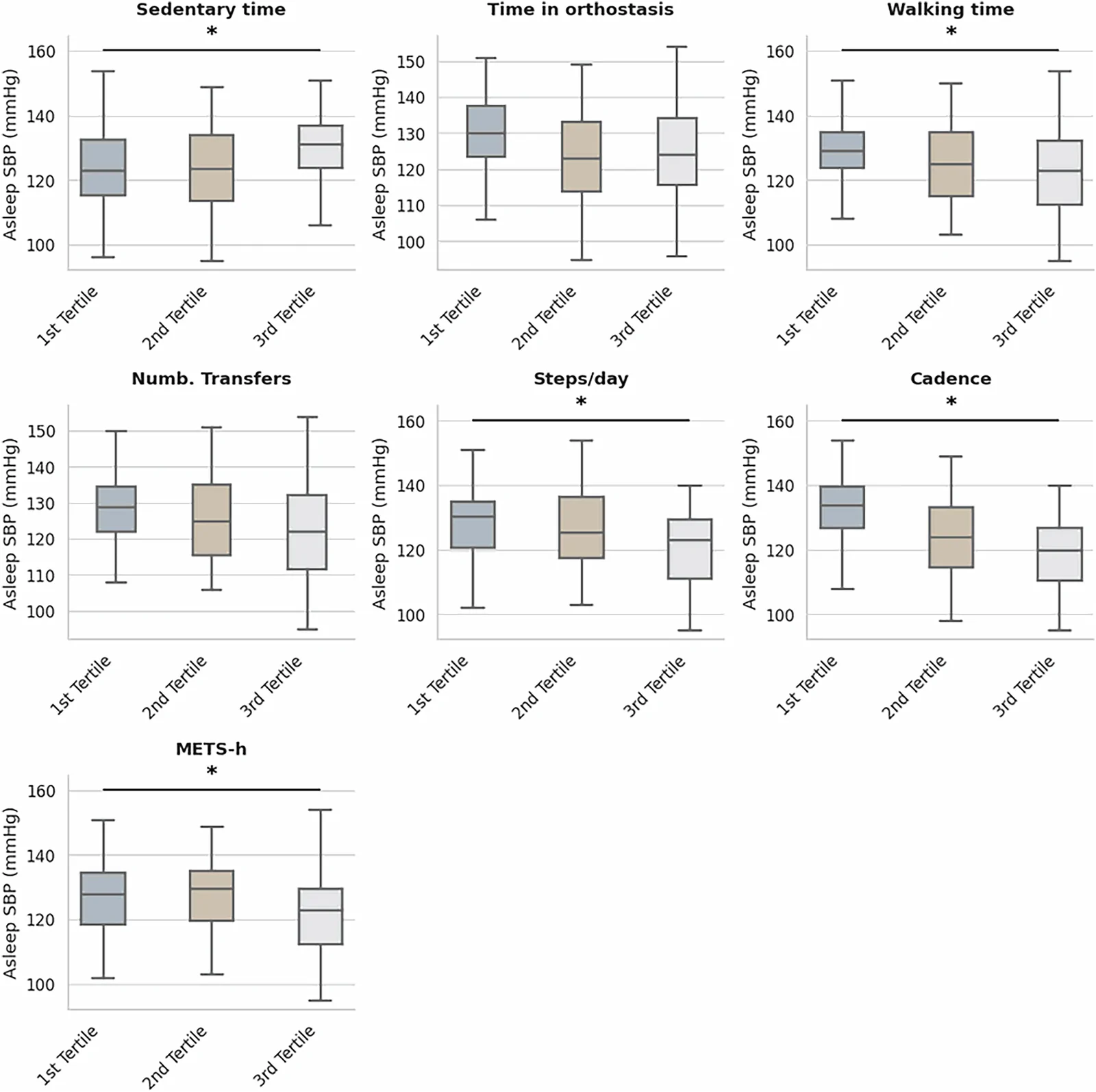
Asleep systolic bood pressure tertiles according to mobility parameters. Boxes represent the median and interquartile range. *P < 0.05 for linear trend.

When participants were stratified by tertiles of mobility parameters, higher sedentary time was associated with elevated nighttime SBP (*p* = 0.046). In addition, lower walking time (*p* = 0.032), fewer steps/day (*p* = 0.012), lower cadence (*p* < 0.001), and lower energy expenditure in METs-h (*p* = 0.033) were all significantly related to higher nighttime SBP [Fig. 2].

**Fig. 3:**
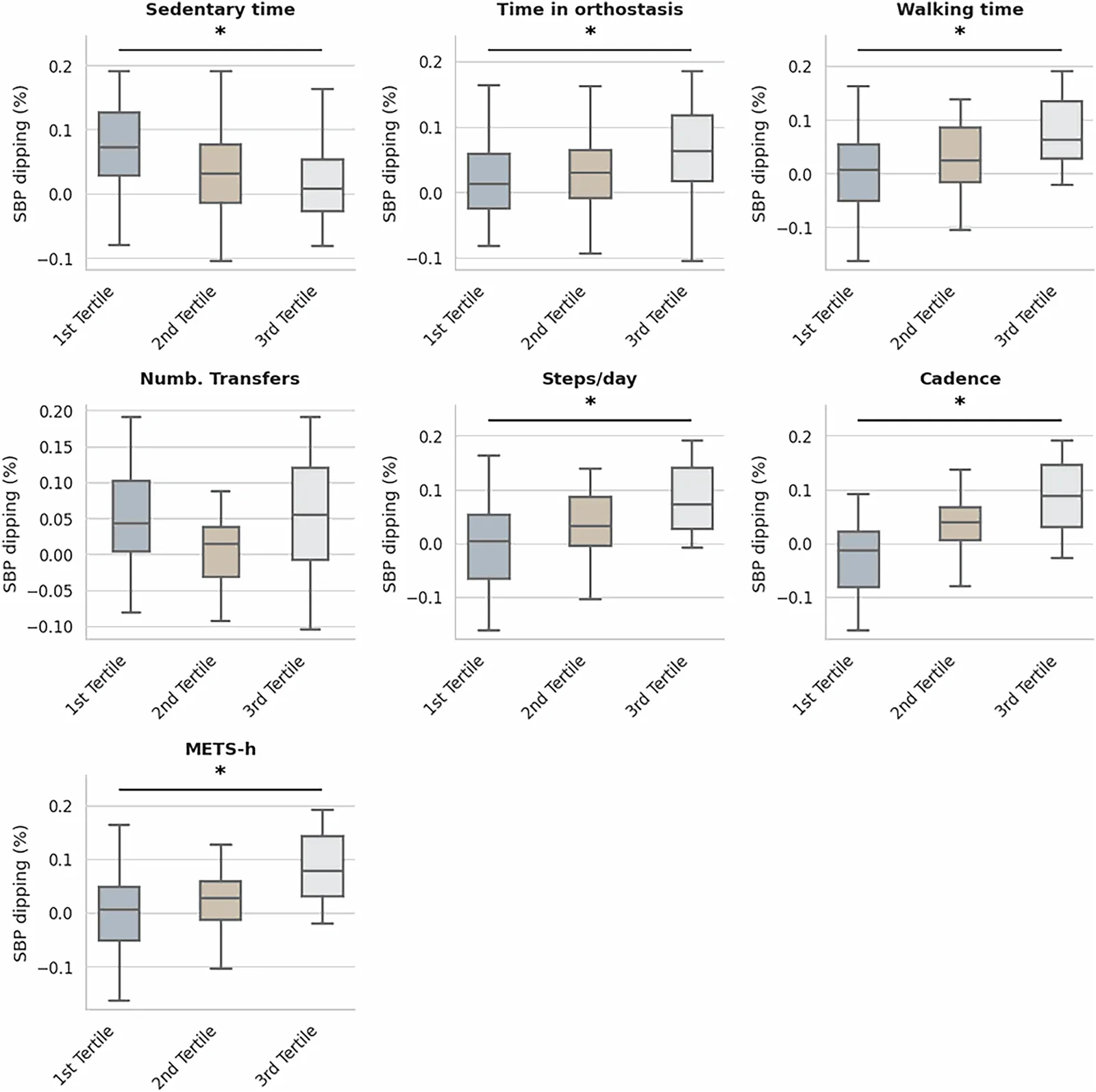
Systolic blood pressure dipping tertiles according to mobility parameters. Boxes represent the median and interquartile range. *P < 0.05 for linear trend.

Regarding nocturnal SBP decline, greater sedentary time was inversely associated with SBP dipping (*p* = 0.004). In contrast, longer walking time (*p* = 0.001), higher steps/day (*p* < 0.001), higher cadence (*p* < 0.001), and higher METs-h (*p* < 0.001) demonstrated positive linear trends with SBP dipping [Fig. 3].

In multivariable logistic regression adjusted for frailty, age, and hypertension, cadence was independently associated with both outcomes. Higher cadence was protective against the non-dipping pattern (OR 0.87, 95% CI 0.79–0.95; *p* < 0.01) and against higher asleep systolic blood pressure (OR 0.56, 95% CI 0.39–0.80; *p* < 0.01). None of the other mobility parameters showed significant associations with these outcomes [Table 3].

**Table 3:** Logistic regression models for the risk of non-dipping and elevated asleep systolic blood pressure.

Variable	OR	CI	p-value
	Nondipping		
Sedentary time (min/day)	0.99	0.9–1.0	0.48
Time in orthostasis (min/day)	1	0.9–1.0	0.55
Walking time (min/day)	0.98	0.96–1.0	0.34
Numb. Transfers	1	0.99–1.0	0.96
Steps/day	1	0.99–1.0	0.14
Cadence (steps/min)	0.87	0.79–0.95	<0.01
METS-h	0.94	0.85–1.0	0.23
	Asleep systolic blood pressure^a^		
Sedentary time (min/day)	0.99	0.97–1.0	0.79
Time in orthostasis (min/day)	1	0.97–1.0	0.75
Walking time (min/day)	0.93	0.83–1.0	0.27
Numb. Transfers	0.99	0.97–1.0	0.44
Steps/day	0.99	0.99–1.0	0.06
Cadence (steps/min)	0.56	0.39–0.8	<0.01
METS-h	0.78	0.45–1.0	0.11

Model adjusted for: frailty, age and hypertension.

*OR* odds ratio, *CI* confidence interval, *SBP* systolic blood pressure.

^a^OR indicates the risk of increasing 1 mmHg in asleep SBP.

## Discussion

In this cross-sectional study of individuals aged 80 years and older, multiple mobility parameters were associated with asleep blood pressure alterations. Higher sedentary time was linked to elevated nighttime SBP and reduced SBP dipping, whereas greater walking time, steps/day, cadence, and energy expenditure were consistently associated with lower nighttime SBP and greater SBP dipping. Among these parameters, cadence emerged as the strongest and most consistent predictor, remaining independently associated with both lower asleep SBP and normal dipping pattern after adjustment for frailty, age, hypertension, and 24-h blood pressure. These findings suggest that objective measures of mobility may provide valuable insights into cardiovascular risk in individuals 80 years and older, particularly in the context of frailty where traditional assessments are often challenging.

In our study, the mean age was 85.3 years, superior to most of observational studies and clinical trials on this topic [20, 21]. The inclusion of frailty on initial assessment favors applicability since its prevalence can be as high as 40% in individuals 80 years and older [17].

Frailty has been associated with asleep blood pressure changes and higher cardiovascular risk, but few studies analyze the role that mobility exerts over this association [9, 22, 23]. Our findings suggest that the strong association between frailty and cardiovascular diseases (CVD) may be related to sedentary lifestyle and low functionality, which are themselves linked to adverse events [23,24]. However, the opposite direction is also plausible, as frailty, cardiovascular disease, and impaired blood pressure regulation may reduce daily activity and favor sedentary behavior. Therefore, these relationships should be interpreted as potentially bidirectional. The biological plausibility of this association is supported by evidence showing that some CVD-related biomarkers (interleukin-6, isoprostanes, and osteoprotegerins) are increased in slow individuals regardless of frailty [25]. This interpretation is also clinically reinforced by studies showing that the relationship between mortality and SBP may be modified according to gait speed [26–28].

Previous studies have consistently demonstrated that sedentary behavior and low physical activity are associated with adverse cardiovascular outcomes, including impaired nocturnal blood pressure regulation [29]. In our study, greater sedentary time was linked to higher nighttime SBP and reduced SBP dipping, whereas higher walking time, steps/day, and energy expenditure in METs-h were protective, showing favorable trends toward preserved nocturnal blood pressure patterns. These findings align with evidence from large epidemiological studies in older adults, which highlight the beneficial effects of even modest increases in daily ambulation and reduced sedentary time on blood pressure control and cardiovascular risk [30]. Importantly, our results extend these observations to individuals over 80 years, a population frequently excluded from prior research, underscoring the relevance of mobility-related behaviors as modifiable determinants of nocturnal blood pressure.

Though frailty is necessary in cardio-geriatric assessment, its evaluation is still diverse, mainly due to the lack of standardized parameters [31]. According to the findings in this study, cadence is presented as a plausible parameter for objective assessment in this population. It showed correlation with the best prognostic markers of future cardiovascular events amongst ABPM findings [8], has easy outpatient applicability and closely corresponds to other mobility parameters.

Cadence represents the number of steps per minute during ambulation and differs conceptually from gait speed. While gait speed is usually obtained in a supervised, standardized walking test and reflects distance covered per unit of time, cadence reflects the stepping rhythm achieved during habitual daily ambulation. For this reason, cadence may provide a practical and objective marker of free-living mobility, especially in very old adults. In our study, cadence was the mobility parameter most consistently associated with asleep systolic blood pressure and systolic dipping, including after multivariable adjustment [32, 33]. Although there is a lack of evidence in the literature, one study showed that cadence had an inverse association with arterial stiffening, which may justify the findings in the current study [34]. Our findings complement the evidence by showing that increased cadence was associated with a lower risk of unfavorable blood pressure measurements in a population where all individuals have arterial stiffening and high cardiovascular risk due to cardiovascular ageing. It also raises a reasonable therapeutic target for future interventions. Prospective studies designed to identify optimal cut-off points are warranted to incorporate cadence as a new risk factor and intervention target in this population.

The strengths of this study include the use of automated devices for measuring mobility and 24-h blood pressure, the elevated mean age, the assessment of frailty in the inclusion criteria and an initial comprehensive assessment. Although other validated methods are available for mobility assessment, many are less feasible for continuous monitoring in routine clinical practice and free-living research settings. Therefore, despite the absence of a single gold standard, actigraphy remains a practical and objective approach for evaluating habitual mobility. Nevertheless, its accuracy may vary according to device type, body placement, algorithm, and walking speed, which may be particularly relevant in very old adults. In addition, although actigraphy has been validated in different populations and settings, validation specifically in adults aged 80 years and older remains more limited.

The small number of patients is the main limitation of this study, as it reduces statistical power. In addition, the exclusion of pre-frail individuals, although methodologically intended to contrast the two extremes of the frailty spectrum in a reduced sample, limits the applicability of our findings to intermediate stages of frailty. The cross-sectional design also limits causal inference.

Hypertension was more prevalent among frail participants, and this variable was included in the adjusted statistical models. However, the timing of medication administration and the specific antihypertensive classes were not analyzed in relation to the outcomes of interest. Although the number of antihypertensive drugs did not differ between groups, medication timing and antihypertensive class may influence asleep blood pressure levels, and these variables were not accounted for in the multivariable analyses. In addition, although sleep-related parameters were assessed in the broader study protocol, they were not formally included in the present sub-analysis. Given the reduced sample size, including additional variables in the multivariable models could have compromised model stability and reduced analytical power. Therefore, the analysis was intentionally focused on mobility, frailty, and ambulatory blood pressure. Nevertheless, the potential influence of sleep fragmentation on the night of ABPM, including any effect related to the monitoring procedure itself, cannot be completely excluded.

In summary, this cross-sectional study demonstrated that multiple mobility parameters were associated with asleep blood pressure alterations in frail and non-frail adults aged 80 years and older. Sedentary time, walking time, steps/day, cadence, and energy expenditure all showed significant trends with nighttime SBP and systolic dipping. Among these, cadence emerged as the most robust and independent predictor, remaining associated with lower asleep SBP and preserved dipping even after adjustment for age, hypertension and frailty status. These findings highlight the relevance of objectively measured mobility parameters in the cardiovascular assessment of adults aged 80 years and older, supporting the use of simple and reproducible markers such as cadence in both frail and non-frail individuals. A concise overview is provided in the Summary Table.

## Summary Table

### What is known about topic

- In adults ≥80 years, asleep systolic blood pressure and loss of the normal dipping pattern are strong markers of cardiovascular risk and mortality, independent of clinic blood pressure.
- Frailty in very old adults is associated with higher prevalence of non-dipping, but frailty scoring is heterogeneous and not always practical in routine cardiology.
- Mobility impairment and sedentary behavior are common in this age group and have been linked to adverse cardiovascular outcomes.

### What this study adds

- Objectively measured mobility was closely associated with asleep systolic blood pressure alterations and systolic dipping in adults aged 80+ years, both frail and non-frail.
- Sedentary status related to higher asleep SBP and reduced dipping, whereas greater walking activity and cadence related to lower asleep SBP and preserved dipping.
- Cadence emerged as the single most robust indicator: higher cadence independently predicted both lower asleep SBP and lower odds of non-dipping, even after adjustment for age, hypertension, and frailty. This suggests cadence may function as a simple, clinically usable risk marker in adults ≥80 years.

## Data Availability

The authors confirm that the data supporting the findings of this study are available within the article. If necessary, additional data are available from the corresponding author, FGB, upon reasonable request.
